# Analytical Modelling and Optimization of the Temperature-Dependent Dynamic Mechanical Properties of Fused Deposition Fabricated Parts Made of PC-ABS

**DOI:** 10.3390/ma9110895

**Published:** 2016-11-04

**Authors:** Omar Ahmed Mohamed, Syed Hasan Masood, Jahar Lal Bhowmik

**Affiliations:** 1 Department of Mechanical and Product Design Engineering, Swinburne University of Technology, Hawthorn 3122, Victoria, Australia; smasood@swin.edu.au; 2 Department of Statistics, Data Science and Epidemiology, Swinburne University of Technology, Hawthorn 3122, Victoria, Australia; jbhowmik@swin.edu.au

**Keywords:** fused deposition modeling (FDM), IV-Optimal response surface design, artificial neural network, process parameters, storage compliance, loss compliance, optimization

## Abstract

Fused deposition modeling (FDM) additive manufacturing has been intensively used for many industrial applications due to its attractive advantages over traditional manufacturing processes. The process parameters used in FDM have significant influence on the part quality and its properties. This process produces the plastic part through complex mechanisms and it involves complex relationships between the manufacturing conditions and the quality of the processed part. In the present study, the influence of multi-level manufacturing parameters on the temperature-dependent dynamic mechanical properties of FDM processed parts was investigated using IV-optimality response surface methodology (RSM) and multilayer feed-forward neural networks (MFNNs). The process parameters considered for optimization and investigation are slice thickness, raster to raster air gap, deposition angle, part print direction, bead width, and number of perimeters. Storage compliance and loss compliance were considered as response variables. The effect of each process parameter was investigated using developed regression models and multiple regression analysis. The surface characteristics are studied using scanning electron microscope (SEM). Furthermore, performance of optimum conditions was determined and validated by conducting confirmation experiment. The comparison between the experimental values and the predicted values by IV-Optimal RSM and MFNN was conducted for each experimental run and results indicate that the MFNN provides better predictions than IV-Optimal RSM.

## 1. Introduction

Fused deposition modelling (FDM) process is one of the most widespread additive manufacturing processes developed by Stratasys that produces three-dimensional (3D) objects by depositing successive cross-sections from feedstock plastic filament [1]. This process begins by designing 3D computer aided design (CAD) model and exporting it into stereolithography (STL) file. Then STL file is imported into the FDM pre-processing software to slice it and create support structures and to set-up the tool path parameters. This process extrudes the plastic filament through a nozzle to form the bottom layer of a part in a controlled way, one layer at a time. The nozzle head moves in both the *X*- and *Y*-axis direction [2]. This process continues until all layers of the part have been completed. The support structures for this process are water-soluble and can be removed manually or by dissolving it out from the manufactured part. A wide variety of industrial thermoplastics can be processed by FDM 3D printing technology such as Polyphenylsulfone (PPSF/PPSU), Polycarbonate (PC), Acrylonitrile-Butadiene-Styrene (ABS), Nylon 12, Acrylonitrile Styrene Acrylate (ASA), and PC-ABS.

Polycarbonate (PC) is a commonly used industrial thermoplastic due to its high performance and distinct properties, including its extremely high heat and dimensional stability, high resistance, low shrinkage, and good mechanical and electrical properties. Acrylonitrile-butadiene-styrene (ABS) is one of the most widely used thermoplastic material in various industrial applications [3]. Usually, the use of PC is limited by its processing properties and mainly low stress applications. It is known that an addition of a small amount of ABS to PC can lead to improved mechanical and processing properties of PC. Because of its economic value and low weight, PC-ABS blend has received significant attention in diverse industrial applications, where high heat resistance and toughness are required [4]. PC-ABS material is being routinely processed with additive manufacturing systems such as FDM Fortus series for direct manufacturing of production components, functional parts and tooling. However, the quality and mechanical properties of PC-ABS parts processed by FDM systems are significantly affected and controlled by the selection of FDM process parameters. These parameters control the final PC-ABS performance and microstructural characteristics of manufactured parts and affect the fiber-to-fiber bonding strength. The dependence of the part properties to the printing parameters provides the manufacturers and production engineers the ability to improve the mechanical performance by adjusting the meso and macro structures of the printed part.

Dynamic mechanical analysis (DMA) is a testing and analytical technique used to characterize the mechanical responses of the sample subjected to periodic dynamic stress in a cyclic manner, mostly sinusoidal deformation force with very small amplitudes [5]. The specimen will then deform to a specific amount. This allows us to measure how much the sample deforms in relation to its stiffness and its response to stress as a function of temperature, frequency and time is to be studied. This technique helps designers to understand and predict the material behaviour for long-term effects under cyclic loading conditions [6]. The temperature-dependent dynamic mechanical properties of samples processed by FDM technology depend on various factors such as slice thickness, raster to raster air gap, deposition angle, part print direction, bead width, and number of perimeters. Dynamic compliance is a characteristic representing how much the sample loses its stiffness under the load effect, which is used for quality control in product development. It is the ratio of peak resultant strain to peak applied stress under cyclic loading conditions. The in-phase component of the dynamic compliance is known as the storage compliance, while the out-of-phase component of the dynamic compliance is known as the loss compliance. A greater compliance values indicate weaker samples and lower compliance indicate stronger samples. The higher the value of dynamic compliance is, the more flexible and less rigid the sample is. Thus, the lower the value of dynamic compliance represents better temperature-dependent dynamic mechanical properties of the processed sample with the FDM process.

Temperature-dependent dynamic mechanical properties are important properties for FDM products, particularly in the aerospace and automotive industry. Practically, this means that the functionality and applicability of a material can change significantly over a wide range of temperatures [7]. This may cause the appearance of defects and loss of functionality of product processed by FDM technology. When using FDM technology for designing and fabrication of structural and functional parts, it is important to understand how FDM technological parameters affect temperature-dependent dynamic mechanical properties of processed products. The degree of change in dynamic mechanical properties over a wide range of temperatures of FDM processed parts depends on many process variables. These variables mainly include slice thickness, deposited angle, part print direction, raster to raster air gap and raster width, which are selected during pre-processing on the FDM software. Structure of the FDM product is formed during melt solidification during the fabrication phase and defines the temperature-dependent properties of the final manufactured part in solid state (i.e., its physical and mechanical properties). The quality and properties of FDM manufactured products are specified by their reliability, functionality and durability [1]. During their use, the FDM manufactured components are repeatedly subjected to different cyclic loading and thermal conditions, which results in increased deformation rates, a change in geometry, and finally failure of product. Because of their importance in industrial applications, it is well worth studying temperature-dependent dynamic mechanical performance of FDM fabricated part from various perspectives. Hence, a wider use of FDM process requires a better understanding of the relationship between the printing conditions and temperature-dependent dynamic mechanical properties and deformation of processed part, which are important for engineering applications.

Recently, a number of experimental studies have been done to optimize the static mechanical properties, surface roughness, dimensional accuracy, and processing time of FDM made parts by selecting a range of proper deposition conditions. For example, Wu et al. [8] studied the effect of Layer thickness and raster angle on the mechanical performance of polyether-ether-ketone (PEEK) samples processed by FDM process. They reported that raster angle and layer thickness have a significant impact on tensile, compressive and bending strength. Murphy et al. [9] developed a novel microcrystalline cellulose (MCC) reinforced polylactic acid (PLA) for FDM applications. They studied the effect of cellulose content on the thermal and dynamic mechanical properties of the biocomposites. Dynamic mechanical analysis results reveal an increase in storage modulus when compared with neat PLA. Mohamed et al. [10] characterized the dynamic mechanical properties of FDM printed samples. The results showed that slice thickness, air gap and number of contours have a marked effect on the part performance. Zou et al. [11] investigated the effect of build orientation on mechanical property. This study concluded that 3D printed materials behave with anisotropic property due to the layer by layer process used in 3D printing technology. Biranchi et al. [12] demonstrated that the slice height and extrusion velocity have great effect on the deformation, while filling velocity and line width compensation have strong effect on the dimensional accuracy of FDM built part. Wendt et al. [13] studied the impact of manufacturing conditions on the tensile and compression strength of FDM processed prototypes. Lanzotti et al. [14] carried out an experimental study on the effect of process parameters on tensile strength of FDM fabricated part by PLA. The results indicated that infill orientation, layer thickness and perimeters have significant impact on the part strength. Rayegani and Onwubolu [15] established relationships between FDM parameters and tensile strength using designed experiment. It was found that the optimum process settings to improve the mechanical properties are negative air gap, small raster road and zero build orientation. Wang et al. [16] reported that the mechanical properties of carbon fiber-reinforced plastic composites are mainly affected by the manufacturing conditions. Impens and Urbanic [17] examined the effect of post-processing parameters on tensile and compression strengths. The study concluded that the part orientation is the most influential factor on the tensile and compression strengths. Ertan [18] investigated the influence of process conditions such as raster angle and build orientation on the mechanical performance and surface roughness of processed parts through FDM. The study revealed that the build orientation is the most significant factor than the raster angle which affects the mechanical properties and surface roughness.

Unfortunately, to date, within the body of available literature, no published studies have investigated the impact of FDM extrusion parameters on temperature-dependent dynamic mechanical properties and deformation behavior of FDM processed parts. The simultaneous as well as individual effect of various fabrication conditions on temperature-dependent dynamic mechanical properties of FDM printed parts is not developed yet. The main reason for the lack of such studies is that experimental techniques for complete characterization of temperature-dependent dynamic mechanical properties are relatively time-consuming. Hence, the aim of this study is to investigate, examine and optimize the FDM printing parameters to improve temperature-dependent dynamic mechanical properties (i.e., storage compliance and loss compliance) of FDM processed PC-ABS parts using response surface methodology (RSM) and multilayer feed-forward neural networks (MFNNs). The IV-optimality response surface design was used to quantify and justify the correlation between the input printing parameters and temperature-dependent dynamic mechanical properties. Subsequently, analysis of variance (ANOVA) technique was employed to test the significance of the printing variables and the developed regression models. The MFNN was applied to predict the storage compliance and loss compliance of the processed part by FDM, as well as to determine the optimal setting of the parameters to achieve minimum storage compliance and loss compliance. Furthermore, failure analysis was analyzed using SEM images. Finally, process parameter optimization was conducted to find optimal printing conditions.

## 2. Experimental Producers

### 2.1. Experimental Work

DMA tests were carried out using a TA Instruments Model 2980 DMA instrument (TA Instruments, New Castle, DE, USA). In this study, 60 specimens of standard geometry of 35 mm in length, 12.5 mm in width, and 3.5 mm in thickness were prepared as per the standard ASTM D4065-01 [19] and also according to the testing procedure specified by the main manufacturer of thermal analysis and rheology instrument (TA Instruments) [20]. Before beginning the tests, the DMA instrument was calibrated using standard specimen (steel compliance calibration beam) based on recommended procedure as specified by DMA machine manufacturer in order to eliminate any clamping impact and effect of DMA instrument compliance. All 60 samples were fabricated by FDM Fortus 400 system (Stratasys, Eden Prairie, MN, USA).

In DMA test, the specimen is mounted in place between the movable and stationary grips (single cantilever configuration). Then, the specimen is placed inside the thermal chamber (furnace) and the periodic dynamic force is applied to the specimen in the moveable clamp which moves slowly over the specified temperature range from 35 to 170 °C. The PC-ABS specimens were heated at a rate of 5 °C/min. A fixed frequency of 1 Hz and displacement amplitude of 15 µm were used for all tests. All tests were carried out in the air atmosphere. Typically, soak time of 300 s is used to ensure that the specimen has reached its thermal equilibrium prior to the sinusoidal load is applied. In this experiment, two types of temperature-dependent dynamic mechanical properties, namely storage compliance (J’) and loss compliance (J’’) of FDM processed samples were measured, analyzed and investigated. The temperature-dependent dynamic mechanical properties of samples processed by FDM technology depend on various factors such as slice thickness, raster to raster air gap, deposition angle, part print direction, bead width, and number of perimeters. All the reported values of storage compliance and loss compliance in this study were calculated as an average from a set of tested specimens for each experimental run as per experimental matrix presented in Table 1. Thermal Advantage software for 2980 DMA instrument is used to manage and analyze the data collected from experiments.

### 2.2. Experimental Design

**Figure 1:**
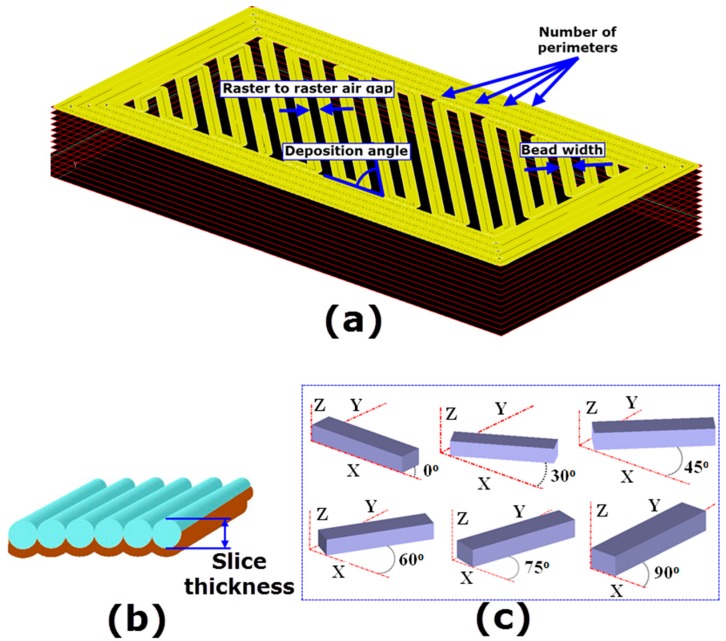
(**a**) Fused deposition modeling (FDM) tool path conditions; (**b**) slice thickness; and (**c**) part print directions.

Response surface methodology (RSM) is an empirical modeling techniques used to explore the relationships between dependent variables and independent variables [21]. In order to establish functional relationships between the printing parameters involved and temperature-dependent dynamic mechanical properties (storage compliance and loss compliance), the experimental plan was carried out based on IV-optimality criterion. “IV” denotes the Integrated Variance over the entire design space. IV-optimality criterion (also known as I-, Q-, and V-optimality) is a class of response surface designs recommend to generate RSM when the main purpose is to find the optimal process conditions and to model the true behavior of the response with greater precision [22]. This is because IV-optimality criterion and its algorithm picks design points that provide minimum integral variance of the prediction across the region of interest. The IV-optimality criterion algorithm proposed 38 experimental runs, consisting of 28 runs of the model points, 5 runs to estimate lack-of-fit and 5 replicates design points, for the 6 printing parameters at different levels. The IV-optimality matrix was augmented with additional design points (22 runs) to check the curvature in the center of the factor region and to reduce the prediction error and to develop regression models with better accuracy of prediction. Table 1 shows the FDM process variable investigated in this study. The printing parameters and their levels are selected based on the past literature and the specifications of the FDM printer manufacturer (Stratasys). The augmented experimental design matrix along with the measured storage compliance and loss compliance is presented in Table 2. Figure 1 shows schematic representation process parameters.

## 3. Results and Discussion

### 3.1. Multiple Regression Analysis

Multiple regression analysis was done based on backward elimination technique. Regression procedure systematically starts with all model variables and then removes the least significant variable (terms with *p* > 0.1) during each step. Backward elimination technique is considered to be the most robust technique for model term reduction as it gives a chance for all model terms to be included in the developed model. The end results from the backward elimination multiple regression analysis is a high-level fitting model for the experimental data with smaller variance than the full model using all variables. In the present study, the quadratic model is used for storage compliance and loss compliance as expressed in Equation (1).

(1) $$Y=\beta_{0}+\overset{k}{\underset{i\;=\;1}{\sum }}\beta_{i}X_{i}+\overset{k}{\underset{i\;=\;1}{\sum }}\beta_{ii}X_{i}^{2}+\;\overset{k}{\underset{i<j}{\sum }}\beta_{ij}X_{i}X_{j}+\varepsilon \;$$

Y is the dependent variable (response), $\beta_{0}$ is the intercept of the regression model, $\beta_{i}$ is the coefficient of the linear terms, $\beta_{ij}$ is the coefficient for interaction terms, $\beta_{ii}$ is the quadratic term of each predictor, $X_{i}$ and $X_{j}$ are the coded independent predictors, and ε is the variance.

The response function representing the output response can be expressed as in Equation (2):

(2) $$Y=f\;\left(X_{1},\;X_{2},\;X_{3},\;X_{4},X_{5},X_{6}\right)+\varepsilon$$

where: $X_{1},X_{2},X_{3},X_{4},\;X_{5},\;\mathrm{and}\;X_{6}$ are slice thickness, raster to raster air gap, deposition angle, part print direction, bead width and number of perimeters, respectively, and ε is the experimental error.

The quadratic model was fitted with the experimental data obtained on the basis of IV-Optimal design. The final IV-Optimal RSM models in terms of actual factors were developed and given below:

(3) $$\begin{array}{ll} \mathrm{Storage}\;\mathrm{compliance} & \\ = & 5.880\times 10^{5}-6.763\times 10^{5}X_{1}+1.222\times 10^{5}X_{2}\\ - & 525.099X_{4}-1.388\times 10^{6}X_{5}\;-40.544X_{6}-399.508X_{2}X_{4}\\ - & 5895.076X_{2}X_{6}+1183.565X_{4}X_{5}+1.264\times 10^{6}X_{1}{}^{2}\\ - & 68033.481X_{2}{}^{2}+1.271\times 10^{6}X_{5}{}^{2} \end{array}$$

(4) $$\begin{array}{ll} \mathrm{Loss}\;\mathrm{compliance} & =-46242.04-3.264\times 10^{5}X_{1}+64709.869X_{2}\\ & +202.814X_{3}-172.091X_{4}+5.587\times 10^{5}X_{5}+79.380X_{6}\\ & +28516.246X_{1}X_{2}+1.112\times 10^{5}X_{1}X_{5}-71.439X_{2}X_{3}\\ & -172.677X_{2}X_{4}-56115.820X_{2}X_{5}-2657.451X_{2}X_{6}\\ & +385.318X_{4}X_{5}+5.230\times 10^{5}X_{1}{}^{2}-1.936X_{3}{}^{2}-5.651\\ & \times 10^{5}X_{5}{}^{2} \end{array}$$

**Figure 2:**
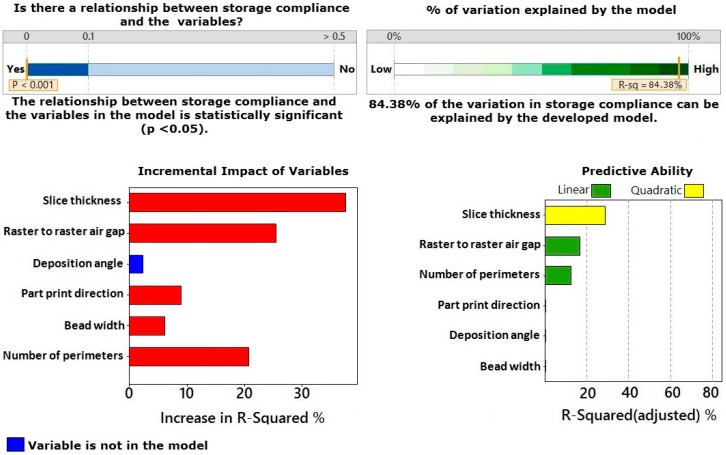
Multiple regression analysis for storage compliance.

Figure 2 shows the multiple regression analysis for the storage compliance. This figure reports that the developed regression model for storage compliance is statistically significant with a *p* value less than 0.001 and has an *R*^2^ value of 84.38%. The results also indicate which factors were included in the model. It can be seen that slice thickness, raster to raster air gap, part print direction, bead width and number of perimeters were included in the developed model to estimate the curvature and their interaction terms. The regression results presented in Figure 2 also show the details about the incremental impact of variables on storage compliance model and the factors contributed to increase the prediction capability of the developed model. Slice thickness, raster to raster air gap, and the number of perimeters are the most significant factors in the developed model, followed by part print direction and bead width. Furthermore, Figure 2 shows the predictive ability of the developed model. The higher values indicate better single predictors of storage compliance. Although deposition angle is a not significant factor, it was included in the model as this variable can improve the predictive capability of the model.

**Figure 3:**
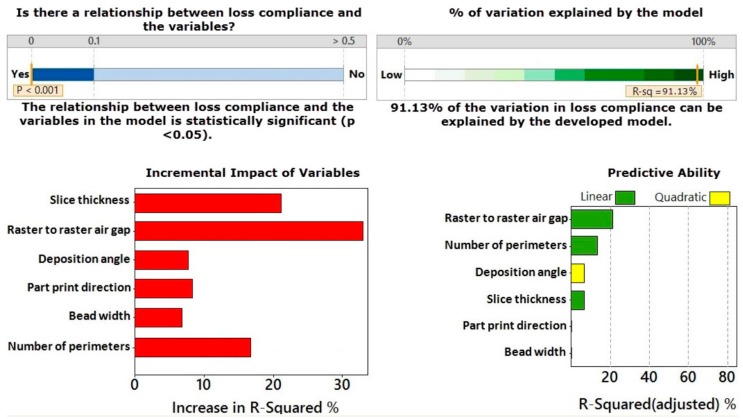
Multiple regression analysis for loss compliance.

Figure 3 illustrates the stepwise regression analysis for the loss compliance. It is clear from this figure that the developed model for the loss compliance is significant as the *p* value is less than 0.001 and the model has an *R*^2^ value of 91.13%. These results indicate that all variables have significant effect on the loss compliance. The most influential factors are raster to raster air gap, slice thickness and number of perimeters, followed by part print direction, deposition angle and bead width. It can also be seen from this figure that raster to raster air gap, number of perimeters and slice thickness are the most important factors that contribute to improve the predictive capability of the developed model in a linear relationship (as can be seen in the green bars). The deposition angle has a strong effect on optimizing the predictive capability of the model as a quadratic term (as can be seen in yellow bar). Therefore, all variables are included in the developed model.

**Figure 4:**
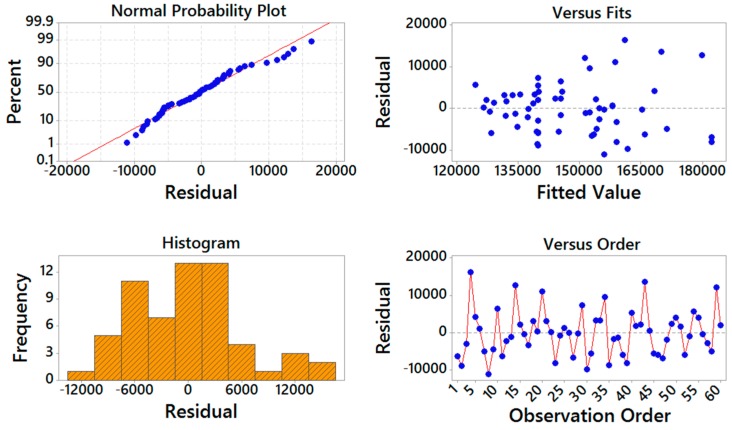
Residual analysis for storage compliance.

**Figure 5:**
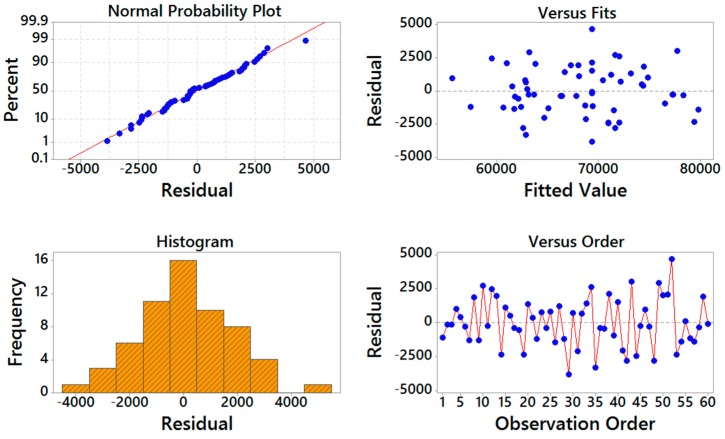
Residual analysis for loss compliance.

Figure 4 and Figure 5 show the residual analysis plots for both storage compliance and loss compliance, respectively. From these figures, it can be seen that the experimental data points follow the straight line, demonstrating that the residuals have a normal distribution. These plots demonstrate that the residuals show a linear pattern, which means the predicted values are well fitted with the actual values. The figures also show that the points are falling randomly on the both sides of zero, suggesting that there are no unusual points that have influence on the response during the experiment.

### 3.2. Influence of Processing Parameters on Storage Compliance and Loss Compliance

**Figure 6:**
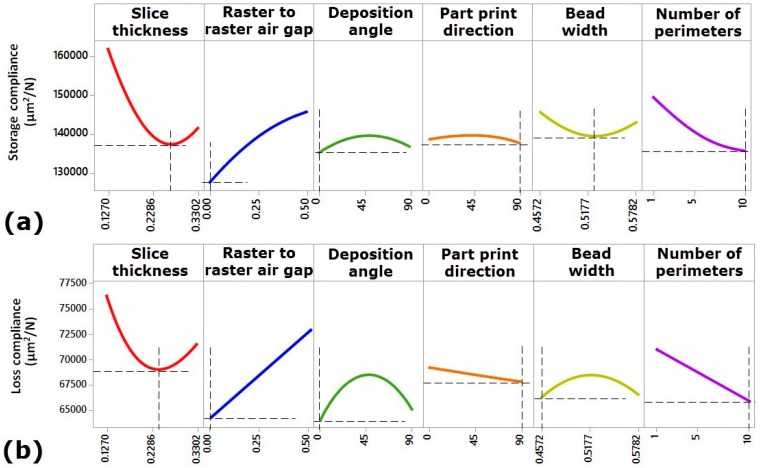
Influence of processing parameters on (**a**) storage compliance; and (**b**) loss compliance.

The effects of each single process parameter on storage compliance (J’) and loss compliance (J’’) were investigated. Results indicate that storage compliance and loss compliance greatly depend on all the investigated parameters. Main effect plot is used to compare the effect of all parameters at particular points of the design space. Main effect plots are presented in Figure 6a,b for the six processing parameters at the central point of the design space. A steep slope shows that storage compliance and loss compliance are sensitive to changes in that variable, while a relatively flat line shows insensitivity. It can also be seen from Figure 6a,b that there are high curvatures in the responses in relation to the process parameters. This helps to pick up the optimal setting of the factor, which is the benefit of having many levels of each variable and this is why IV-Optimal design is optimal. It is evident from Figure 6a,b that slice thickness is one of the factors that have the greatest influence on dynamic compliance. With the increase in slice thickness, a remarkable nonlinear reduction in storage compliance and loss compliance was observed up to a certain level of slice thickness (0.2540 mm). This is because the lower setting of slice thickness increases the number of layers, which are necessary to print the final product. This can increase the micro-voids formation between two layers at the interface of the molten layers. This creates air entrapment, thereby increase porosity. It can be seen from Figure 6a,b, the slice thickness of 0.2540 mm provides the minimum values of dynamic compliance. However the further increase in slice thickness would tend to increase in the storage compliance and loss compliance. The reason behind this increase in the storage compliance and loss compliance between 0.2540 to 0.3302 mm is that higher values of slices create thick rasters and layers, which cannot fill up the small sub perimeter voids; hence porosity increases in the interior regions of the part. Furthermore, the stair-stepping effect increases with the increase in slice thickness, which leads to brittle structure of the printed part due to unfilled space formed. Therefore, the value of 0.2540 mm for slice thickness provides uniform thick rasters and layers. Thus, the processed part with slice thickness of 0.2540 mm tends to be more flexible and stiff to the applied load.

**Figure 7:**
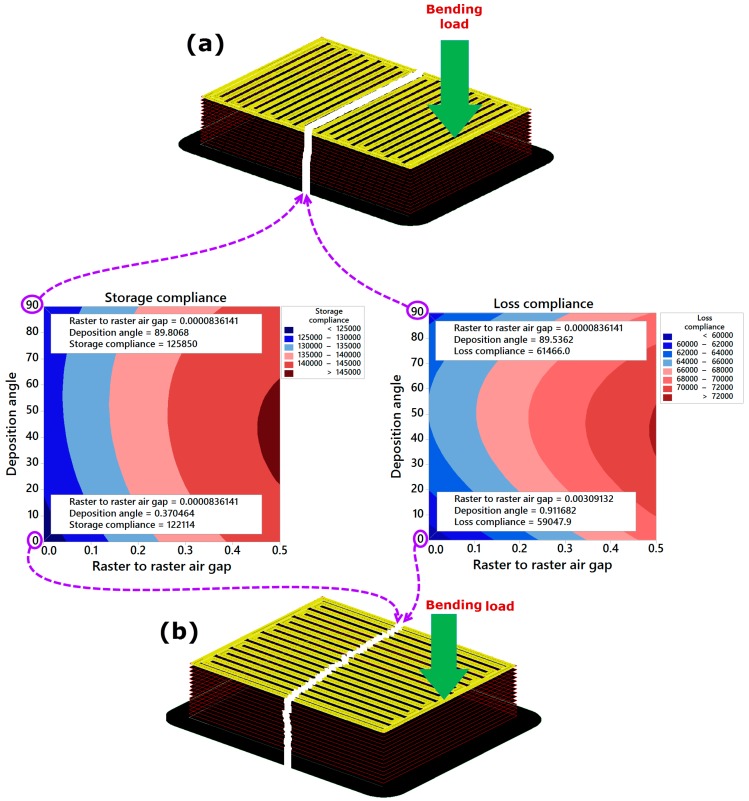
Failure analysis of deposition angle on the (**a**) part printed with deposition angle of 0°; and (**b**) part printed with deposition angle of 90°.

The effect of raster to raster air gap is significant on either storage or loss compliance of the printed part. The storage compliance or loss compliance was observed to systematically increase with increasing raster to raster air gap (see Figure 6a,b). This result is expected because zero raster to raster air gap creates a slight overfill of the part and increases its resistance to the deformation under cyclic loading conditions. However, zero raster to raster air gap has a consequential effect on the product aesthetics because of overfilling at stacked regions which results in uneven layer, and thus undesirable quality of the surface. While, if the positive value of raster to raster air gap is used then there would be spacing between rasters, hence it will create a more aesthetically pleasing part and reduce build time and material consumption, but the storage or loss compliances get increased. As a result, the processed part with the positive value of raster to raster air gap tends to be weaker and it may not recover its pristine dimension and position thoroughly after the dynamic force is released. Deposition angle has a marginal effect on the storage compliance, but it has a noticeable influence on the loss compliance. As it is shown in Figure 6a,b, by increasing the value of deposition angle from 0° to 45°, the mean of the storage compliance increased slightly, but significant increase in loss compliance was observed. On further increase of deposition angle beyond 45°, either storage compliance or loss compliance of FDM processed part starts decreasing. This is due to the fact that deposition angle of 45° increases the number of rasters compared to the deposition angles of 0° and 90°. Many input rasters tend to promote stress accumulation along the direction of the material deposition, leading to more distorted structure of the processed part responsible for weaker layer bonds. According to ES-Said et al. [23], the anisotropy properties in FDM processed part are produced by the polymer molecules align themselves with the direction of flow, creation of pores in part microstructure and weak interlayer bonding. For a better illustration of the effect of different deposition angles on the part properties and performance, Figure 7 shows three different specimens processed with two deposition angles (0° and 90°) and subject to bending load. It is clear from Figure 7 that the bending load requires to fracture longer polymer chains in case of using 0° deposition angle. However, it requires breaking shorter fibers in case of using 0°, as the fracture in this case occurs at the interfacial regions of a bonded rasters. Thus, the processed part with 90° of deposition angle can fail at early stages of applying cyclic loading. Therefore, it can be concluded that the processed part with deposition angle of 0° is stronger than other parts processed with deposition angles of 45° and 90°. This is a useful conclusion which will help additive manufacturing practitioners in understanding the effect mechanism of deposition angle and creating optimal process planning.

Part print direction has a marginal influence on the dynamic compliance as shown in Figure 6a,b. As shown in Figure 6a,b, storage compliance increases up to 45° print direction and then decreases with an increase in print direction. However, loss compliance decreases linearly with the increase in print direction. It is clear that the longest length of the sample oriented towards *Y*-axis exhibits the lowest (with higher mechanical properties) values of storage compliance and loss compliance. This is because in *Y*-axis, by 90° the polymeric chains are oriented forming at 90° on the longest length of the sample with adhesive deposition orientation and tie chains are oriented parallel to the bending load applied on the specimen, creating a stronger part strength to the deformation [8]. Figure 6a,b also illustrates the point that there are conflicting influences of bead width on storage compliance and loss compliance, as the minimum value of storage compliance is obtained using center value of bead width, while the minimum value of loss compliance can be obtained at lower value of bead width (0.4572 mm). The storage compliance can be improved with 0.52 mm of bead width as it provides thick ratsers and provides uniform temperature gradients throughout the fabrication process and hence the processed sample tends to have the highest maximum stored elastic energy. While improved loss compliance is possible with using the lowest bead width value (0.4572) as it provides thinner roads and rasters, which covers up the unfilled regions of the part curves and leads to more ductile sample to energy dissipated. Number of perimeters has a large effect on both storage compliance and loss compliance (see Figure 6a,b). Number of perimeters fills the part curves with outer and inner contours with internal raster fills. It can be seen from Figure 6a,b that the storage compliance and loss compliance can be improved significantly by setting the maximum number of perimeters (10 perimeters). The number of perimeters improves the storage compliance and loss compliance respectively in several ways: (i) the maximum number of perimeters (10 perimeters) minimizes the number of built rasters and reduces the rasters length. Thus, it eliminates voids and unfilled regions of the part curves; (ii) The maximum number of perimeters (10 perimeters) produces stronger perimeter part walls and solid filled regions of the part curves, resulting in the overall high density and increase in the deformation resistance of the built part; (iii) By increasing the number of perimeters, more cyclic loads can be carried over by polymeric chains, and it distributes the loads on wider area along the longitudinal and transverse axis instead of carrying the applied loads by part rasters, resulting in avoidance of premature breakdown of processed parts under cyclic loading conditions.

### 3.3. Interaction Effects

**Figure 8:**
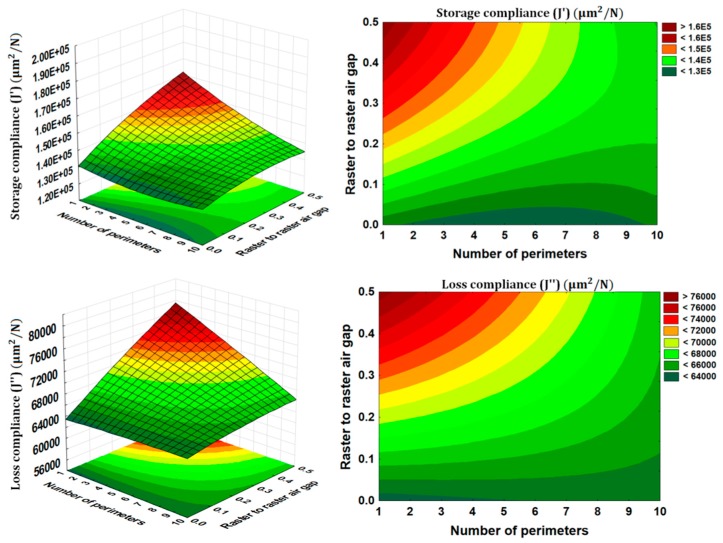
Interaction effects of raster to raster air gap and number of perimeters.

**Figure 9:**
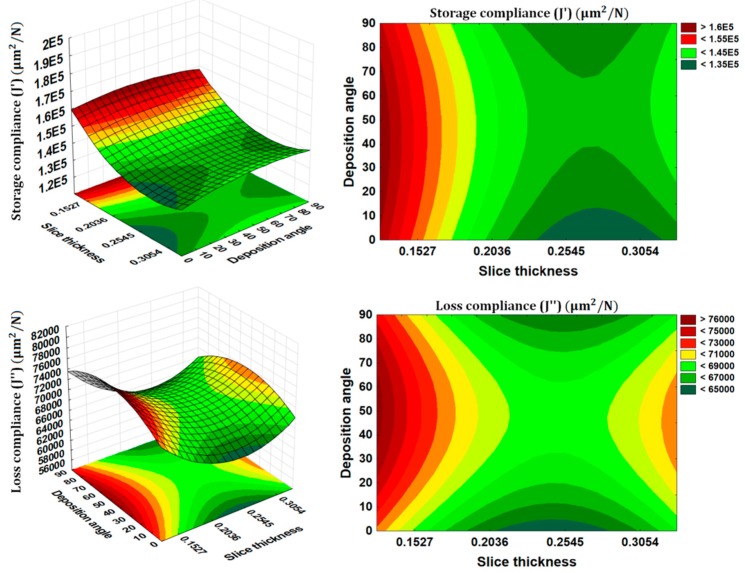
Interaction effects of deposition angle and slice thickness.

The interaction effects as presented in Figure 8 provide us more insight into the influence of the processing parameters on the storage compliance and loss compliance. The interactions effect between raster to raster air gap and the number of perimeters are found to be interesting. As discussed earlier the positive value of raster to raster air gap makes the part weaker than zero raster to raster air gap. However, it appears from Figure 8 that the storage compliance and loss compliance can also be improved using positive value of raster to raster air gap but with combination of maximum number of perimeters (10 perimeters). These combinations of two parameters not only improve the storage compliance and loss compliance, but also reduce the production time and material consumption, as the positive value of raster to raster air speed up the manufacturing process. Therefore, the additive manufacturing users can now use this optimal combination of parameters to produce multifunctional products. Another useful interaction effect was between the deposition angle and slice thickness where, deposition angle having a more significant effect on loss compliance (see Figure 9). This suggests that the smaller deposition angle and slice thickness near 0.2540 mm value are the optimal combinations for both the storage compliance and the loss compliance, while being more sensitive to the loss compliance. All of these results give us insight of the practical aspects of FDM printing process, as well as the physical phenomena involved behind the effect of such processing conditions on the temperature-dependent dynamic mechanical properties.

### 3.4. Morphology

**Figure 10:**
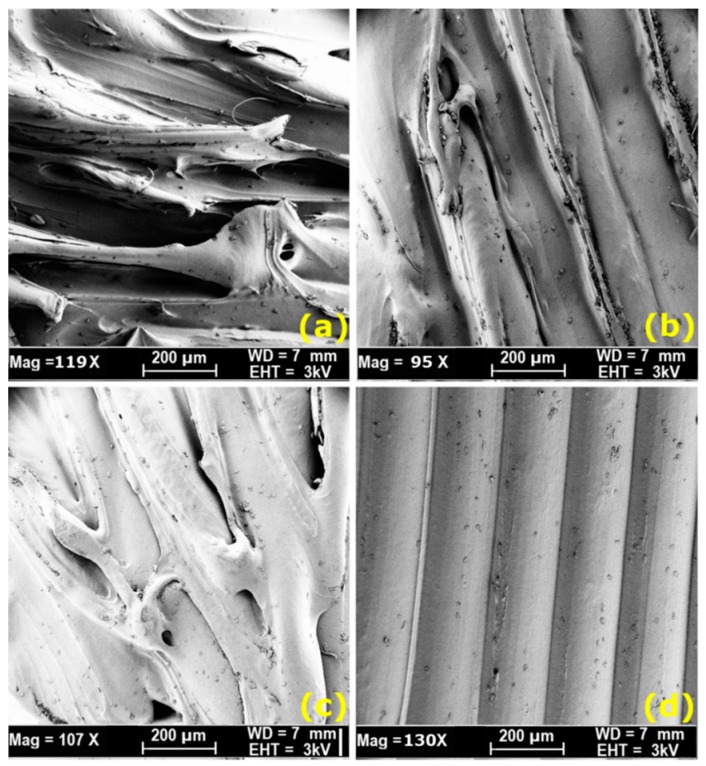
Micrographs of the surface for Polycarbonate-Acrylonitrile-Butadiene-Styrene (PC-ABS) specimens processed with: (**a**) slice thickness of 0.1270 mm; (**b**) slice thickness of 0.1778 mm; (**c**) single perimeter; and (**d**) 10 perimeters, respectively.

The scanning electron microscopy (SEM) observations further provide supporting evidence of trends mentioned in the previous section. SEM (ZEISS SUPRA 40VP FESEM, Carl Zeiss Microscopy, LLC, Oberkochen, Germany) is used to examine and characterize the morphology and microstructure of failed FDM specimens. The specimens were prepared by mounting them on metal stubs and then gold sputter-coated for 60 s prior to SEM viewing, imaging and taking pictures at various magnifications. The SEM micrographs of samples fabricated at a relatively low slice thicknesses (0.127 and 0.1778 mm) are shown in Figure 10a,b respectively. According the results presented in Figure 10a, the sample processed with slice thickness of 0.127 mm can show surface defects such as voids, lamellar tearing, and delamination. While results presented in Figure 10b confirms that by increasing the value of slice thickness from 0.127 to 0.1778 mm, a visible improvement in the sample surface and structure cab be observed with minimum voids and without too much lamellar tearing and delamination. These results are in agreement with the results presented in the main effect plots in Figure 6a,b. Figure 10c shows the sample fabricated with a single perimeter (1 perimeter), while Figure 10d shows the micrographs of the surfaces of PC-ABS specimen, which was fabricated with maximum number of perimeters (10 perimeters) along with a fixed level of other parameters. After comparing the micrographs between Figure 10c for specimen fabricated with a single perimeter and Figure 10d for specimen fabricated with 10 perimeters, a visible difference in the part structure can be observed. The sample printed with 10 perimeters showed high density, indicating more dense regions. As a consequence, the presence of the maximum number of perimeters in the samples resulted in stronger microstructure and more rigid sample to the deformation. The specimen processed with a single perimeter presented weaker bonded rasters and pores and delamination structure as shown in Figure 10c, because a single perimeter pass may leave an air gap because of increasing the number of rasters, particularly if the perimeter width is not large enough for a raster fill. Thus, the samples fabricated with a single perimeter breaks easily and reduce its deformation strength to the bending load.

### 3.5. Modeling with Multilayer Feed-Forward Neural Network (MFNN)

**Figure 11:**
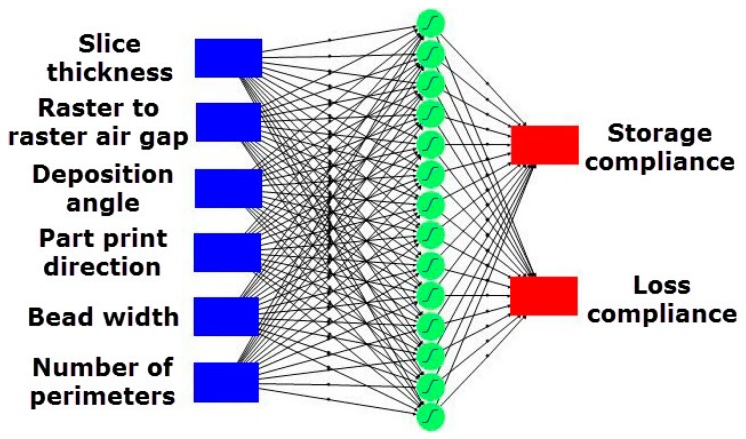
Schematic diagram of the artificial neural network (ANN) model developed to predict the storage compliance and loss compliance.

**Figure 12:**
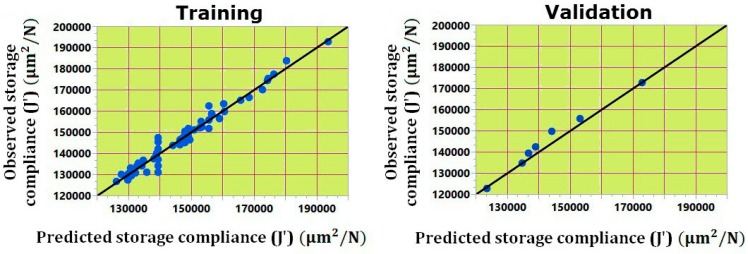

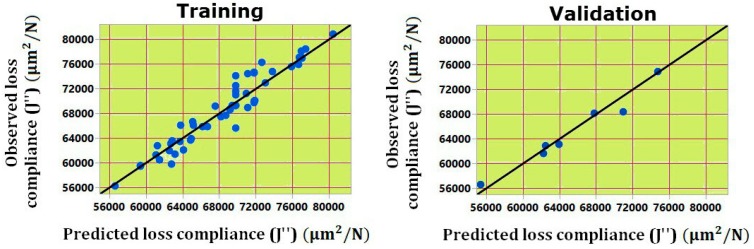
Scatterplots for predicted versus observed values for the storage compliance and loss compliance.

Artificial neural network (ANN) is a computational technique inspired by the function of a biological network used to solve complex and nonlinear problems in various applications [24]. The construction of an ANN consists of input layer, hidden layer and output layer. In this study, a multilayer feed-forward neural network (MFNN) model was developed using layered feed-forward ANNs namely, multilayer perceptron (MLP) with sigmoidal function. In MLP, the neurons are structured in layers, and send the signals “forward”. The value of errors is then propagated backwards through the network by neurons in the input layer [25]. This error signal is used to adjust the values of weight coefficients, and the output of the network is then given through the neurons on an output layer. The first step in this type of ANN modeling is to optimize the neural network to obtain ANN architecture with minimal errors and dimension in data training and testing. The experimental data obtained though the IV-optimality RSM was used to build effective ANN models and to feed the network in order to train it. In this study, the neural network architecture used is presented in Figure 11. This form of the neural network architecture has six inputs (slice thickness, raster to raster air gap, deposition angle, part print direction, bead width and number of perimeters) and two outputs (storage compliance and loss compliance). In the present study, the optimum number of 14 neurons in hidden layers was selected upon maximizing *R*^2^ and minimizing the difference between predicted values by ANN model and the actual values. The *k*-fold method is used as a cross validation method with total number of 5 folds [26]. In the training of ANN, *k*-fold cross validation method is used to make the result more reliable. In *k*-fold cross validation method, a part of experimental data is used for training ANN network to estimate model parameters, while other experimental data those were not included in the training process were used to assess the predictive capability (hidden data) of the model. The MFNN described in this study is used to predict the storage compliance and loss compliance of the processed part by FDM, as well as to determine the optimal setting of the parameters to achieve minimum storage compliance and loss compliance. The optimal setting of the parameters is evaluated through desirability function. The goodness of fit between the predicted values obtained by the ANN model and the experimental values were used to check the ANN model performance. It can be observed from Figure 12 that the ANN model is predicting on the experimental data that were not used to train the model, as the predicted values obtained by ANN model are strongly correlated with the experimental values and the ANN model has *R*^2^ value of 96.39% and 95.11% for storage compliance and loss compliance, respectively.

#### Comparison between ANN and IV-Optimal RSM Models

**Figure 13:**
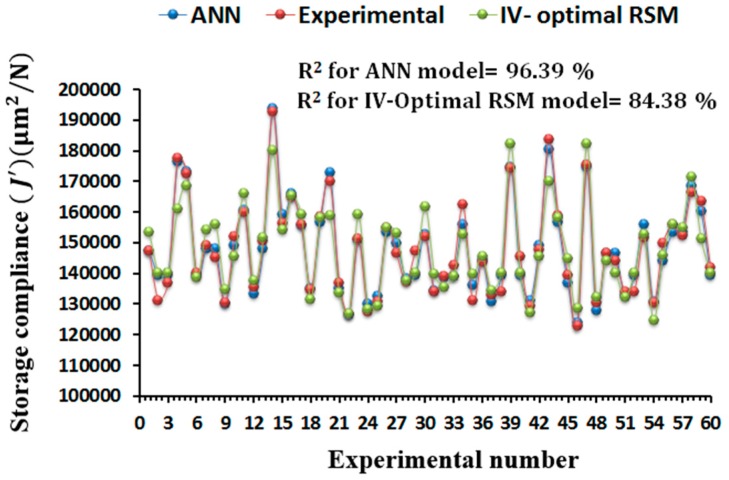
Comparison of experimental results with predicted values obtained by the ANN and IV-optimal response surface methodology (RSM) model for the prediction of storage compliance.

**Figure 14:**
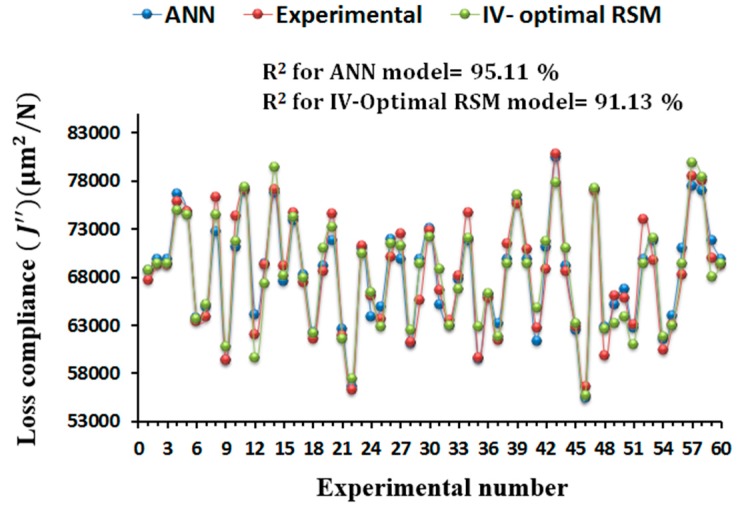
Comparison of experimental results with predicted values obtained by the ANN and IV-optimal RSM model for the prediction of loss compliance.

The ANN and IV-Optimal RSM based predictive models for the storage compliance and loss compliance were compared with the experimental values in terms of the predictive capability as shown in Figure 13 and Figure 14. The values obtained by ANN and IV-Optimal RSM are compared for 60 runs of experiments presented in IV-Optimal design matrix. It is clear from Figure 13 and Figure 14 that the variation in predictive values for the storage compliance and loss compliance is very little, but slightly more variations in IV-Optimal RSM models than in the ANN models. A strong correlation between experimental values and predicted values was noticed with maximum prediction error of less than 2%. The ANN models performed slightly better because of the high nonlinearity relationships between the process parameters and storage compliance and loss compliance. However, IV-Optimal RSM models also predict the similar nonlinear relationships between the variables very well. This confirms the applicability of the ANN and IV-Optimal RSM in the prediction and optimization of FDM processing conditions, with the minimum experimental runs at multiple levels of each variable, particularly when compared with traditional experimental designs such as central composite design when the replications of design points is included.

**Figure 15:**
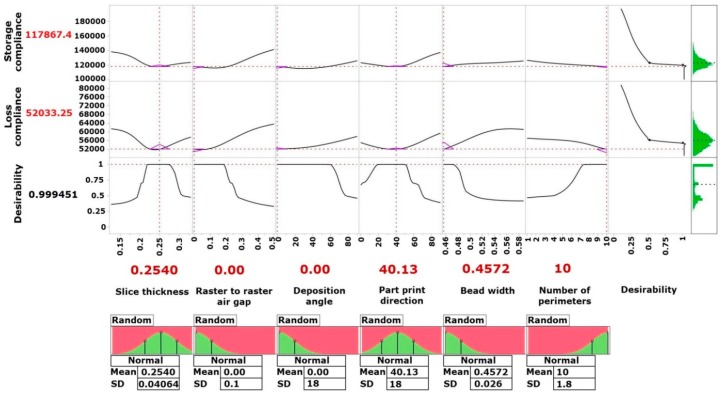
Desirability profiling and optimization.

Figure 15 illustrates the desirability profiling and optimization with sensitivity indicator for the optimal setting of processing parameters determined through the ANN models. This is useful in case of large parameters with multiple responses to be able to quickly screen the sensitive cells. In order to optimize the process parameters, the following constraints are considered:

0.1270 mm ≤ $X_{1}$ ≤ 0.3302 mm
0 ≤ $X_{2}$ ≤ 0.5 mm
0° ≤ $X_{3}$ ≤ 90°
0° ≤ $X_{4}$ ≤ 90°
0.4572 mm ≤ $X_{5}$ ≤ 0.5782 mm
1 ≤ $X_{6}$ ≤ 10

The overall desirability has a scale between zero and one (least to most desirable respectively) which can be defined as the geometric mean of the desirabilities obtained for each response. The overall desirability value of “1” signifies the ideal case and a value of “0” represents that one or more responses are outside the acceptable limits. From Figure 15, it is obvious that the optimum process parameters were found to be slice thickness of 0.2540 mm, zero raster to raster air gap, disposition angle of 0°, part print direction of 40.13°, bead width of 0.4572 mm and 10 perimeters. At this optimum process conditions, storage compliance and loss compliance reduction were found to be 117,867.4 and 52,033.25 μm^2^/N, respectively, with overall desirability value of 0.999451. Confirmation experiment was conducted at this optimum condition and the results obtained from the confirmation experiments are presented in Table 3. The results clearly indicate that the proposed ANN model and augmented IV-optimality RSM have the modeling competence and prediction accuracy. In addition, the suitability and applicability of the developed models are justified.

## 4. Concluding Remarks

This paper has presented an investigation of the impact of the process conditions on the temperature-dependent dynamic mechanical properties of processed PC-ABS parts by FDM technology. FDM process conditions have been found to play a significant role in optimizing the dynamic mechanical properties as a function of temperature. The IV-optimality response surface was used to develop regression models to create functional relationships between input parameters (slice thickness, raster to raster air gap, deposition angle, part print direction, bead width, and number of perimeters) and output variables (storage compliance and loss compliance). Adequacy of the developed models and its respective predictors was validated by the multiple regression analysis along with the statistical graphs and confirmation experiment. Based on the experimental results, the following conclusions are drawn:

- This study has shown for first time that the FDM process conditions have significant influence on the temperature-dependent dynamic mechanical properties of printed PC-ABS parts.
- This work has proposed an effective approach to improve the dynamic mechanical properties as of the FDM fabricated parts as a function of temperature by accurately selecting suitable multi-level process parameters with less number of experiments compared to the traditional response surface designs such as central composite design and face centered composite design when considering the replication of design points.
- The IV-optimality response surface design was found to be an efficient and effective design in process optimization involving many parameters with multiple levels.
- Although MFNN performed slightly better, the IV-Optimal RSM was also found to be a promising design in prediction performance, where good agreement between IV-Optimal RSM models, MFNN models and experimental results was observed.
- It has been observed that storage compliance is more sensitive to slice thickness, raster to raster air gap, and the number of perimeters followed by part print direction and bead width. However, the deposition angle is less effective.
- It has been found that loss compliance is significantly affected by the variables. However, raster to raster air gap, slice thickness and number of perimeters are found to be the most influential variables.
- All parameters can be used effectively for improvement in the dynamic mechanical properties as a function of temperature. The anisotropic behavior of FDM built part was mainly caused by interlayer porosity and weak interlayer bonding.
- Based on multi-response optimization process, slice thickness of 0.2540 mm, no raster to raster air gap, disposition angle of 0°, part print direction of 40.13°, bead width of 0.4572 mm and 10 perimeters seem to be the favourable values to improve the temperature-dependent dynamic mechanical properties of the parts printed by FDM.

## Figures and Tables

**Table 1:** Selected fused deposition modeling (FDM) factors and their levels.

Factor	Symbol	Unit	Level 1	Level 2	Level 3	Level 4	Level 5	Level 6
Slice thickness	$X_{1}$	mm	0.1270	0.1778	0.2540	0.3302	-	-
Raster to raster air gap	$X_{2}$	mm	0	0.1	0.2	0.3	0.4	0.5
Deposition angle	$X_{3}$	Degree	0	15	30	45	60	90
Part print direction	$X_{4}$	Degree	0	30	45	60	75	90
Bead width	$X_{5}$	mm	0.4572	0.4814	0.5056	0.5298	0.5540	0.5782
Number of perimeters	$X_{6}$	-	1	3	5	7	8	10

**Table 2:** IV-Optimal design plan and measured temperature-dependent mechanical properties. “S.” means “sequence”.

S. No	Factors						Responses		S. No	Factors						Responses	
	$X_{1}$	$X_{2}$	$X_{3}$	$X_{4}$	$X_{5}$	$X_{6}$	$J^{\prime }$ $\left(\mu m^{2}/N\right)$	$J^{\prime \prime }$ $\left(\mu m^{2}/N\right)$		$X_{1}$	$X_{2}$	$X_{3}$	$X_{4}$	$X_{5}$	$X_{6}$	$J^{\prime }$ $\left(\mu m^{2}/N\right)$	$J^{\prime \prime }$ $\left(\mu m^{2}/N\right)$
1	3	6	6	6	1	1	147,118	67,605.5	31	4	6	4	3	1	6	134,002	66,648.3
2	3	4	4	3	4	3	131,033	69,235.0	32	4	6	6	6	6	6	138,809	63,518.3
3	3	4	4	3	4	3	136,947	69,235.0	33	4	6	1	1	6	6	142,436	68,142.8
4	4	5	6	2	2	1	177,380	75,885.9	34	4	3	5	6	6	1	162,230	74,662.6
5	1	3	4	6	1	1	172,573	74,839.4	35	3	6	1	6	6	4	131,088	59,555.3
6	3	3	4	6	1	3	139,907	63,371.1	36	4	3	1	1	6	1	143,774	65,864.4
7	1	1	1	3	1	3	149,156	63,814.7	37	4	1	1	4	6	3	133,017	61,386.3
8	4	6	1	6	2	1	144,983	76,276.9	38	3	4	4	3	4	3	134,008	71,511.7
9	4	1	6	6	1	5	130,321	59,417.7	39	1	6	1	6	6	1	174,213	75,588.2
10	1	1	4	1	3	4	151,821	74,398.9	40	3	4	4	3	4	3	145,352	70,910.0
11	4	6	6	1	6	1	159,770	77,059.7	41	3	1	4	6	3	4	129,203	62,674.9
12	4	3	1	6	1	6	135,250	62,019.3	42	1	1	4	1	3	4	147,701	68,863.5
13	4	4	6	1	1	4	150,393	69,223.7	43	2	6	4	2	6	1	183,669	80,813.4
14	2	6	1	1	1	1	192,771	77,087.9	44	1	3	4	3	6	6	158,671	68,532.9
15	1	1	6	3	1	6	156,206	69,187.4	45	1	1	1	1	6	1	139,286	62,858.5
16	1	6	6	5	3	3	164,988	74,728.0	46	3	1	1	1	1	6	122,608	56,563.3
17	1	6	6	1	6	6	155,824	67,458.0	47	1	4	6	1	1	1	175,489	76,955.9
18	4	1	6	4	2	1	134,693	61,586.0	48	3	4	6	2	3	6	130,149	59,779.1
19	1	3	4	3	6	6	158,461	68,621.9	49	2	1	1	6	6	6	146,428	66,077.9
20	1	6	3	5	5	5	169,847	74,528.4	50	2	4	6	6	4	6	144,053	65,860.6
21	4	1	3	1	1	1	136,729	61,905.2	51	3	2	6	2	6	3	133,867	63,086.1
22	3	6	6	6	1	6	126,739	56,229.2	52	3	4	4	3	4	3	134,081	74,051.5
23	1	1	6	6	6	2	151,075	71,195.6	53	4	3	5	6	6	1	151,642	69,696.0
24	4	1	3	3	4	6	127,379	66,034.9	54	4	1	6	1	6	6	130,200	60,367.1
25	2	1	6	1	4	1	130,522	63,574.9	55	3	4	1	3	1	3	149,788	63,106.9
26	3	6	6	5	5	1	154,905	70,040.4	56	1	4	1	3	3	6	155,763	68,279.9
27	2	6	5	1	1	6	146,426	72,460.2	57	4	6	4	1	3	3	152,173	78,448.7
28	2	6	1	6	3	1	137,239	61,210.1	58	1	5	2	4	3	2	166,293	78,017.9
29	3	4	4	3	4	3	147,252	65,559.7	59	1	6	2	6	1	6	163,460	69,954.7
30	1	4	1	1	4	4	152,018	72,879.2	60	3	4	4	3	4	3	141,899	69,298.1

**Table 3:** Results of confirmation experiment.

Dependent Variables	Optimal Process Settings						Actual Values	IV-Optimal RSM Model at 95% CI	ANN Model
	$X_{1}$	$X_{2}$	$X_{3}$	$X_{4}$	$X_{5}$	$X_{6}$			
Storage compliance	0.2540	0	0	40.13	0.4572	10	118,046.2	122,925	117,867.4
Loss compliance							53,334.03	53,562.7	52,033.25

## References

[1] Mohamed O.A., Masood S.H., Bhowmik J.L. (2015). Optimization of fused deposition modeling process parameters: A review of current research and future prospects. Adv. Manuf..

[2] Chapman B. Increasing Toughness of 3D-Printed Plastic Using Acetone Vapor. https://benchapman4.wordpress.com/2014/05/08/increasing-toughness-of-3D-printed-plastic-using-acetone-vapor/.

[3] Whelan A. (2012). Polymer Technology Dictionary.

[4] Ferry J.D. (1980). Viscoelastic Properties of Polymers.

[5] Menard K.P. (2008). Dynamic Mechanical Analysis: A Practical Introduction.

[6] Murayama T. (1978). Dynamic Mechanical Analysis of Polymeric Material.

[7] Arivazhagan A., Masood S. (2012). Dynamic mechanical properties of abs material processed by fused deposition modelling. Int. J. Eng. Res. Appl..

[8] Wu W., Geng P., Li G., Zhao D., Zhang H., Zhao J. (2015). Influence of layer thickness and raster angle on the mechanical properties of 3D-printed PEEK and a comparative mechanical study between PEEK and ABS. Materials.

[9] Murphy C.A., Collins M.N. Microcrystalline cellulose reinforced polylactic acid biocomposite filaments for 3D printing. Polym. Compos..

[10] Mohamed O.A., Masood S.H., Bhowmik J.L., Nikzad M., Azadmanjiri J. (2016). Effect of process parameters on dynamic mechanical performance of fdm PC/ABS printed parts through design of experiment. J. Mater. Eng. Perform..

[11] Zou R., Xia Y., Liu S., Hu P., Hou W., Hu Q., Shan C. (2016). Isotropic and anisotropic elasticity and yielding of 3D printed material. Compos. Part B Eng..

[12] Panda B.N., Shankhwar K., Garg A., Jian Z. (2016). Performance evaluation of warping characteristic of fused deposition modelling process. Int. J. Adv. Manuf. Technol..

[13] Wendt C., Fernández-Vidal S.R., Gómez-Parra Á., Batista M., Marcos M. (2016). Processing and quality evaluation of additive manufacturing monolayer specimens. Adv. Mater. Sci. Eng..

[14] Lanzotti A., Grasso M., Staiano G., Martorelli M., Pei E., Campbell R.I. (2015). The impact of process parameters on mechanical properties of parts fabricated in PLA with an open-source 3-D printer. Rapid Prototyp. J..

[15] Rayegani F., Onwubolu G.C. (2014). Fused deposition modelling (FDM) process parameter prediction and optimization using group method for data handling (GMDH) and differential evolution (DE). Int. J. Adv. Manuf. Technol..

[16] Ning F., Cong W., Hu Y., Wang H. (2016). Additive manufacturing of carbon fiber-reinforced plastic composites using fused deposition modeling: Effects of process parameters on tensile properties. J. Compos. Mater..

[17] Impens D., Urbanic R. (2015). Assessing the impact of post-processing variables on tensile and compression characteristics for 3D printed components. IFAC PapersOnLine.

[18] Durgun I., Ertan R. (2014). Experimental investigation of FDM process for improvement of mechanical properties and production cost. Rapid Prototyp. J..

[19] Active Standard ASTM D4065 (2001). Standard Practice for Determining and Reporting Dynamic Mechanical Properties of Plastics.

[20] (2002). DMA2980 Dynamic Mechanical Analysis.

[21] Myers R.H., Montgomery D.C., Anderson-Cook C.M. (2009). Response Surface Methodology: Process and Product Optimization Using Designed Experiments.

[22] Douglas C.M. (2001). Design and Analysis of Experiments.

[23] Es-Said O., Foyos J., Noorani R., Mendelson M., Marloth R., Pregger B. (2000). Effect of layer orientation on mechanical properties of rapid prototyped samples. Mater. Manuf. Process..

[24] Towell G.G., Shavlik J.W. (1994). Knowledge-based artificial neural networks. Artif. Intell..

[25] Pal S.K., Mitra S. (1992). Multilayer perceptron, fuzzy sets, and classification. IEEE Trans. Neural Netw..

[26] Refaeilzadeh P., Tang L., Liu H. (2009). Cross-validation. Encyclopedia of Database Systems.

